# CD40 Generation 2.5 Antisense Oligonucleotide Treatment Attenuates Doxorubicin-induced Nephropathy and Kidney Inflammation

**DOI:** 10.1038/mtna.2015.40

**Published:** 2015-12-01

**Authors:** Aaron J Donner, Steve T Yeh, Gene Hung, Mark J Graham, Rosanne M Crooke, Adam E Mullick

**Affiliations:** 1 Antisense Drug Discovery, Isis Pharmaceuticals, Inc., Carlsbad, California, USA

**Keywords:** antisense, CD40, inflammation, kidney

## Abstract

Preclinical and clinical data suggest CD40 activation contributes to renal inflammation and injury. We sought to test whether upregulation of CD40 in the kidney is a causative factor of renal pathology and if reduction of renal CD40 expression, using antisense oligonucleotides (ASOs) targeting CD40, would be beneficial in mouse models of glomerular injury and unilateral ureter obstruction. Administration of a Generation 2.5 CD40 ASO reduced CD40 mRNA and protein levels 75–90% in the kidney. CD40 ASO treatment mitigated functional, transcriptional, and pathological endpoints of doxorubicin-induced nephropathy. Experiments using an activating CD40 antibody revealed CD40 is primed in kidneys following doxorubicin injury or unilateral ureter obstruction and CD40 ASO treatment blunted CD40-dependent renal inflammation. Suborgan fractionation and imaging studies demonstrated CD40 in glomeruli before and after doxorubicin administration that becomes highly enriched within interstitial and glomerular foci following CD40 activation. Such foci were also sites of ASO distribution and activity and may be predominately comprised from myeloid cells as bone marrow CD40 deficiency sharply attenuated CD40 antibody responses. These studies suggest an important role of interstitial renal and/or glomerular CD40 to augment kidney injury and inflammation and demonstrate that ASO treatment could be an effective therapy in such disorders.

## Introduction

Approximately 13% of the US population suffers from chronic kidney disease and the prevalence of this condition is expected to rise.^1,2^ Glomerulonephritis is a common manifestation of chronic kidney disease and is associated with chronic interstitial inflammation and fibrosis.^3^ Patients with the most severe manifestations of glomerulonephritis, such as steroid-resistant nephrotic syndrome and recurrent focal segmental glomerulosclerosis (FSGS), are not adequately treated with current therapies and are at high risk for end-stage renal disease.

The CD40-CD40L signaling pathway was initially shown to have a crucial role in lymphocyte-dependent adaptive immunity.^4^ Since those early findings, an appreciation of this pathway in chronic inflammatory diseases has contributed to a much wider understanding of CD40 biology.^5,6,7,8,9,10^ The broad cellular expression of CD40 on nonhematopoietic cells within the kidney like fibroblasts, endothelium, and epithelial cells underscores its role in the regulation of both adaptive and innate immunity in various disease states.^11,12,13,14^

Inhibition of the CD40-CD40L signaling pathway has proven beneficial in multiple rodent models of kidney disease.^15,16,17,18,19,20^ Additionally, recent work has demonstrated that patients at risk of recurrent FSGS have high plasma titers of CD40 autoantibodies which produce renal injury in mice.^21^ These studies suggest that CD40 inhibition would have therapeutic value in the treatment of glomerulonephritis and related renal diseases. Efforts to inhibit CD40-CD40L signaling in patients using antagonistic CD40L antibodies have been stymied by adverse thromboembolic events.^22,23^ Though an antibody-based approach is still being pursued,^24^ antisense oligonucleotides (ASOs) represent an alternative therapeutic approach.

Due to their unique pharmacokinetic distribution into organs such as the kidney, a CD40 ASO could provide the specificity to inhibit renal CD40 with minimal inhibitory activity at sites of poor ASO distribution such as lymphocytes. Such a targeting strategy would be essential in treating chronic CD40-dependent inflammatory conditions without compromising adaptive immunity. Additionally, a CD40 ASO would not be predicted to exhibit any adverse, generalized thrombotic effects nor have partial agonistic or lymphocyte-depleting activities, which are still hurdles to overcome with any antibody-based CD40-CD40L inhibitor.

Generation 2.5 antisense molecules represent the most potent and advanced chemical class of ASOs and are comprised of 2'-4' constrained ethyl (cET) modified bicyclic nucleic acid oligonucleotides flanking a central DNA gap. This modification has previously demonstrated robust kidney activity in rodents and nonhuman primates.^25^ Herein are studies characterizing the renal inflammatory response to CD40 activation in healthy and injured kidneys and an evaluation of the efficacy of CD40 ASO treatment to protect the kidney against such stimuli. These data demonstrate that the renal cortex is a primary site of CD40 signaling which becomes amplified in injured kidneys. Cortical sites of CD40 activation also proved to be excellent sites for ASO activity, as CD40 ASO treatment blunted renal inflammatory responses to CD40 activation and mitigated kidney injury produced by doxorubicin.

## Results

### Mouse kidneys display elevated basal CD40 expression, which is highly upregulated within the cortical interstitium following CD40 activation

**Figure 1:**
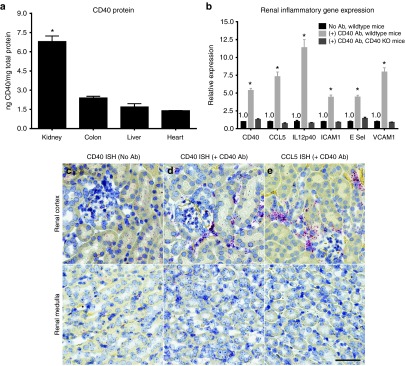
**Kidneys demonstrate high basal CD40 expression, which is highly upregulated within the cortical interstitium following CD40 activation**. (**a**) A comparison of whole organ CD40 protein measured by enzyme-linked immunosorbent assay in healthy, inflammation-naive C57BL/6 mice. (**b**) CD40 and CD40-dependent inflammation measured by quantitative real-time polymerase chain reaction in the kidney 24 hours following IV administration of an activating CD40 mAb in wild-type or CD40-deficient mice. Data are normalized to mice not receiving the Ab. Renal cortex and medulla CD40 *in situ* hybridization (ISH) (**c,d**) and CCL5 ISH (**e**) before administration of the CD40 mAb (**c**) or 24 hours following the CD40 mAb (**d,e**). Mean ± SEM, *n* = 4/group, scale bar = 50 μm, *P < 0.05 versus all tissues (**a**) or versus no Ab control (**b**).

An organ-wide comparison of CD40 mRNA expression in wild-type C57BL/6 mice demonstrated three- to fivefold greater CD40 protein levels in the kidney relative to the colon, liver, or heart (**Figure 1a**). Next, an activating anti-CD40 monoclonal antibody (CD40 mAb) was administered intravenously (IV) and CD40-dependent inflammation in the kidneys was evaluated. Twenty-four hours following antibody-dependent CD40 activation, kidney CD40 mRNA and CD40-dependent inflammation were increased from 4- to over 10-fold and remained unchanged in CD40 knockout mice (**Figure 1b**). Using *in situ* hybridization (ISH), basal CD40 expression was detected within glomeruli and cortical tubular epithelial cells (**Figure 1c**) but was absent in the medulla. Twenty-four hours after CD40 mAb administration, CD40 was dramatically increased at focal sites within the cortical interstitium, including areas within and associated with glomeruli (**Figure 1d**). ISH of CCL5 was also strongly induced in a CD40-dependent fashion and demonstrated a similar pattern of expression (**Figure 1e**).

The feasibility of targeting kidney cortical interstitial cells was evaluated using a Generation 2.5 ASO targeting Malat1. Malat1 is a noncoding nuclear retained RNA that is ubiquitously and highly expressed in the kidney, thereby making it an ideal target to evaluate suborgan ASO activity. Kidneys harvested from mice treated 4 weeks with 100 mg/kg/week Malat1 ASO were evaluated for ASO activity (Malat1 ISH) and distribution (ASO immunohistochemistry (IHC)). Malat1 kidney expression in phosphate buffered saline (PBS)-treated mice (**Supplementary Figure S1a**) was sharply reduced in mice following Malat1 ASO treatment (**Supplementary Figure S1b**). In addition to the robust Malat1 ASO activity within tubular epithelial cells, ASO activity and distribution were also observed at sites within the cortical interstitium (arrows in **Supplementary Figure S1b**,**c**) and glomerulus.

### A CD40 ASO demonstrated potent *in vitro* inhibition of CD40 and CD40-dependent inflammation

**Figure 2:**
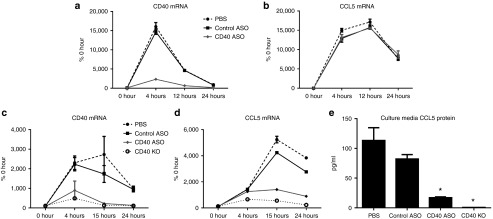
***In vitro* incubation of thioglycollate-elicited peritoneal macrophages with CD40 antisense oligonucleotide (ASO) reduced CD40 and CD40-dependent inflammation**. To evaluate CD40 ASO activity following a broad induction of inflammatory pathways, macrophages were incubated with 10 μmol/l of ASO for 1 hour, washed and then cells were harvested 4, 12, and 24 hours following 0.5 μg/ml lipopolysaccharide and (**a**) CD40 and (**b**) CCL5 quantitative real-time polymerase chain reaction (qRT-PCR) were performed. To evaluate CD40 ASO activity following CD40-dependent inflammation, macrophages were incubated with 10 μmol/l of ASO for 1 hour, washed and then cells were harvested 4, 12, and 24 hours following 100 ng/ml IFN-γ + 10 μg/ml of an activating CD40 mAb and (**c**) CD40 and (**d**) CCL5 qRT-PCR were performed. (**e**) Media removed from the ASO-treated cells exposed to IFN-γ and the CD40 mAb treatments were used for enzyme-linked immunosorbent assay determination of CCL5. Mean ± SEM, *n* = 3/treatment and timepoint, *P < 0.05 versus phosphate buffered saline control.

A lead CD40 ASO was identified by *in vitro* and *in vivo* screens for activity and tolerability as described in the Materials and Methods. This CD40 ASO was further evaluated under the conditions of “free uptake” (*i.e.*, no lipid transfection and/or electroporation) in thioglycollate-elicited peritoneal macrophages (TG-MΦ) in two different screens (**Figure 2**). In the first, CD40 ASO specificity was demonstrated by reduction in CD40 mRNA (**Figure 2a**) and the lack of changes in lipopolysaccharide (LPS)-dependent, CD40-independent increases in CCL5 (**Figure 2b**). The second screen used a combination of IFN-γ to prime CD40 followed by administration of the activating CD40 mAb. As shown in **Figure 2c**,**d**, CD40 mAb-dependent activation of CD40 resulted in a greater than 20- and 50-fold induction of CD40 and CCL5 mRNA, respectively. In contrast, CD40 ASO treatment reduced the induction of CD40 by up to 90% and attenuated CD40 mAb-dependent induction of CCL5 by up to 75%. CCL5 protein levels in the media were analyzed by enzyme-linked immunosorbent assay (ELISA) at 24 hours and were reduced 80% with CD40 ASO treatment (**Figure 2e**). The control ASO demonstrated little to no effect in both screens and no effects of the CD40 mAb were observed in TG-MΦ isolated from CD40 knockout mice.

### CD40 ASO treatment resulted in robust inhibition of CD40 mRNA and CD40-dependent inflammation in the kidney

**Figure 3:**
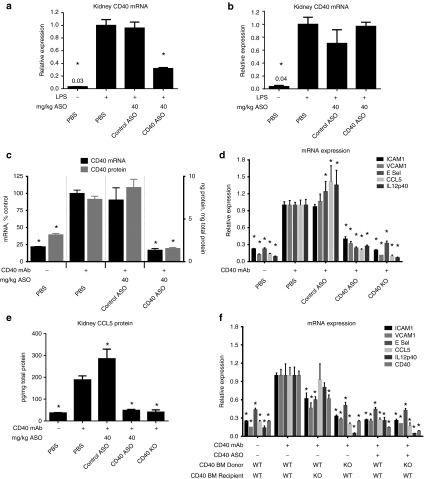
***In vivo* administration of a CD40 antisense oligonucleotide (ASO) resulted in robust reduction of kidney CD40 and CD40-dependent inflammation**. (**a,b**) To evaluate CD40 ASO activity following a broad induction of inflammation, C57BL/6 mice were given 40 mg/kg/week of ASO treatments for 4 weeks followed by 0.5 mg/kg lipopolysaccharide (LPS) given 4 hours before kidneys were harvested. (**a**) Kidney CD40 and (**b**) CCL5 expression as measured by quantitative real-time polymerase chain reaction (qRT-PCR). (**c–e**) To evaluate CD40 ASO activity following CD40-dependent inflammation, C57BL/6 mice were given 40 mg/kg ASO treatments on days 0 and 7, followed by 25 μg of the activating CD40 mAb on day 10 and then kidneys were harvested on day 11 and (**c**) kidney CD40 was measured by enzyme-linked immunosorbent assay (ELISA) and qRT-PCR, (**d**) ICAM1, VCAM1, E Selectin, CCL5 and IL12p40 were measured by qRT-PCR, and (**e**) kidney CCL5 was measured by ELISA. (**f**) WT and CD40 KO recipient mice were irradiated with 7 Gy and then given IV ~400K bone marrow (BM) donor cells of the specific genotype. Seven weeks following reconstitution, animals received two SC administrations of 40 mg/kg ASO (study days 0 and 7) and then they received 25 μg of the activating CD40 mAb (on day 10). Kidneys were harvested on day 11 for mRNA analysis of CD40 and CD40-dependent inflammation. For all datasets, kidney CD40 or CCL5 protein was measured by ELISA and mRNA expression measured by qRT-PCR. Data are reported as relative expression to (+) CD40 Ab, phosphate buffered saline (PBS) group = 1.0, unless otherwise noted. Mean ± SEM, *n* = 4–6/group, *P < 0.05 versus (+) CD40 Ab, PBS group (**a–e**) or versus (+) CD40 Ab, (−) CD40 ASO, WT/BM:WT (**f**).

The CD40 ASO was subsequently evaluated in models of acute kidney inflammation. CD40 ASO treatments were given SC at 40 mg/kg/week for 4 weeks and kidneys were harvested 4 hours following an IP challenge of 0.5 mg/kg LPS (**Figure 3a**,**b**). Previous studies have established maximal kidney CD40 mRNA induction 4 hours following LPS (data not included). As observed in TG-MΦ, CD40 mRNA, but not CCL5 mRNA, was reduced with CD40 ASO treatment and there was no effect with a control ASO. These data add confidence that the CD40 ASO used in these studies did not impact inflammatory pathways unrelated to CD40.

Next, the ability of the CD40 ASO to attenuate CD40-dependent renal inflammation was evaluated in mice given the activating CD40 mAb. Mice received two SC administrations of 40 mg/kg/week ASO (study days 0 and 7) and then they received 25 μg of the activating CD40 mAb (on day 10) by IV. Kidneys were harvested 24 hours after Ab administration. Kidney CD40 protein and mRNA were reduced 80% with CD40 ASO treatment, but were unchanged in mice receiving the control ASO (**Figure 3c**). The activating CD40 mAb produced large elevations in CD40-dependent inflammation and had no effect in CD40 knockout mice (**Figures 1b** and **3d**). Mice receiving the CD40 ASO also displayed 60–80% attenuation of several CD40-dependent inflammatory genes (**Figure 3d**). Although the control ASO had no effect on CD40 expression, it did elevate E Selectin, CCL5, and IL12p40 (IL12/IL23) expression in this one experiment. However, these results were not recapitulated in another study (see below).

A dose–response experiment was performed to further corroborate CD40-dependent ASO activity as well as to determine an optimal dose for pharmacology studies. Mice received 5, 15, or 40 mg/kg of the CD40 ASO or 40 mg/kg of the control ASO on study days 0, 4, and 7. Next, 25 μg of the CD40 mAb was given on study day 10 and then kidneys were harvested 24 hours afterward. Dose-dependent reductions of CD40 and CD40-dependent inflammation were observed with CD40 ASO treatments with little to no changes observed with control ASO treatment (**Supplementary Figure S3a**). Interestingly, markers of macrophages (F4/80), B cells (CD19) and T cells (CD3d) were not altered following acute CD40 activation with the CD40 mAb (**Supplementary Figure S3b**). These data suggest doses above 15 mg/kg would be expected to produce robust kidney CD40 inhibition.

### Bone marrow CD40 deficiency resulted in near complete mitigation of CD40-dependent renal inflammation

To determine the contribution of parenchymal renal cells versus cells of hematopoietic origin, bone marrow transplantation was used to create chimeric mice with CD40 deficiency either on bone marrow or non-bone marrow-derived cells. CD40 KO recipient mice received bone marrow (BM) from wild-type (WT) mice (CD40 KO/BM:WT) whereas the WT recipient mice received BM from either WT or CD40 KO animals (WT/BM:WT and WT/BM:CD40 KO, respectively). Mice received two SC administrations of 40 mg/kg/week ASO (study days 0 and 7) and then they received 25 μg of the activating CD40 mAb (on day 10). Kidneys were harvested 24 hours later and mRNA expression of ICAM1, VCAM, E Selectin, CCL5, IL12p40, and CD40 were analyzed (**Figure 3f**). In the kidneys of WT/BM:WT animals, each of these inflammatory markers were induced by two- to sevenfold following CD40 mAb administration. Relative to WT/BM:WT mice, the induction of CCL5 and IL12p40 were unchanged in CD40 KO/BM:WT mice, but were almost completely abrogated in the WT/BM:CD40 KO mice suggesting myeloid CD40 to be the principal source of CD40-dependent renal cytokine induction. The adhesion molecules ICAM1, VCAM, and E Selectin were induced only 40–50% in CD40 KO/BM:WT mice suggesting a non-BM-derived cell type, such as an endothelial cell, also responds to CD40 activation. CD40 ASO treatment in WT/BM:WT mice resulted in reduced renal inflammation, which approached that observed in mice with myeloid CD40 deficiency. Collectively, these data suggest myeloid-cell-derived CD40 to be the principal cell type that is activated to produce renal inflammation following CD40 mAb administration and further demonstrates the effectiveness of CD40 ASO treatment to blunt renal CD40 activation by targeting myeloid-derived cells.

### CD40 ASO treatment improved renal function and pathology in mice with DOX nephropathy

To explore the role of CD40 in a model of renal disease, we evaluated CD40 ASO treatment in DOX nephropathy. Exploratory studies characterizing the response to 10.5 mg/kg IV DOX administration revealed large variations in animal health and the severity of nephropathy. Therefore, experiments were performed to identify appropriate biomarkers to minimize cohort response variability. Reductions in body weight and increases in urine NGAL and proteinuria were found to be excellent predictors of DOX nephropathy when assessed 1 week following DOX administration and were used to remove outliers. Mice demonstrating a moderate response to DOX were then randomized into four groups and treated weekly over 8 weeks with PBS, control ASO (25 mg/kg), and low dose (12.5 mg/kg), or high dose (25 mg/kg) CD40 ASO. Treatments were initiated 2 weeks after DOX administration to model a therapeutically relevant treatment regimen as previous studies have demonstrated glomerular hypertrophy and significant CD4+ and CD8+ renal cell infiltrates in this model 2 weeks after DOX administration.^26^

**Figure 4:**
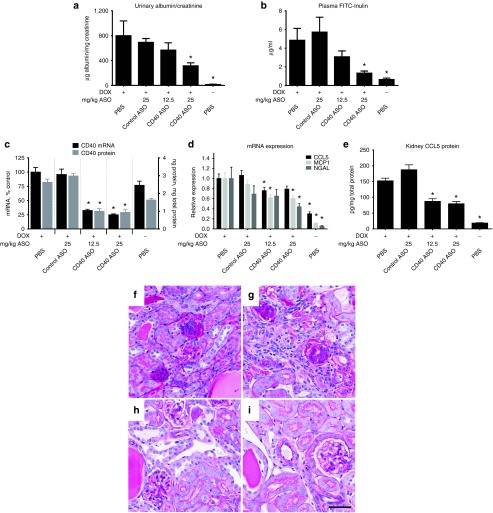
**CD40 ASO treatment mitigated DOX nephropathy**. CD40 antisense oligonucleotide (ASO) and control ASO treatments were initiated 2 weeks following initiation of DOX nephropathy and after 8 weeks of ASO treatments renal functional assessments of (**a**) proteinuria (graphed as the ratio of the urine albumin to creatinine) and (**b**) plasma fluorescein isothicyanate conjugated inulin (FITC)-inulin elimination (assessed by a single measurement of residual plasma FITC-Inulin two hours following FITC-inulin injection) were performed. Additionally, kidneys were harvested and whole kidney (**c**) CD40 mRNA and protein, (**d**) CCL5, MCP-1 and NGAL mRNA, and (**e**) CCL5 protein were measured. (**f–i**) Periodic acid-Schiff staining of representative kidney sections from (**f**) phosphate buffered saline (PBS), (**g**) control ASO, (**h**) 12.5 mg/kg CD40 ASO, and (**i**) 25 mg/kg CD40 ASO groups. Mean ± SEM, *P < 0.05 versus Dox PBS group, *n* = 10–12/group (DOX mice) or 6/group (healthy control mice), scale bar = 50 μm.

Dose-responsive reductions of kidney CD40 and improvements in fluorescein isothicyanate conjugated inulin (FITC-inulin) plasma elimination and proteinuria were observed in mice after receiving 8 weeks of CD40 ASO treatments (**Figure 4a**–**c**). It was noteworthy that FITC-inulin plasma elimination in the 25 mg/kg CD40 ASO group approached that measured in healthy mice. Proteinuria was also maximally reduced in the mice receiving 25 mg/kg of the CD40 ASO. Consistent with reductions of CD40 expression and improvements in kidney function, markers of renal injury, inflammation and fibrosis, like CCL5, MCP-1 and NGAL (**Figure 4d**,**e**) and CTGF (**Supplementary Table S1**), were also reduced with CD40 ASO treatment. Kidney periodic acid-Schiff sections revealed marked improvement in DOX-mediated pathology as demonstrated by an improvement of glomerular nephropathy, interstitial and mesangial expansion and granular tubular casts in CD40 ASO-treated mice (**Figure 4h**,**i**) versus controls (**Figure 4f**,**g**).

It has been demonstrated that T-cells and macrophages have a large influence on disease progression in DOX nephropathy and that CD40L inhibition reduced leukocyte infiltrates.^20^ Consistent with those reports, CD40 ASO treatment decreased renal T-cell recruitment as assessed both by CD3d mRNA expression and CD3d IHC and CD4 and CD8 mRNA expression (**Supplementary Table S1**). Concordant with the reduction in T-cells, IL-2 expression was maximally reduced in the high-dose CD40 ASO group. We did not observe a decrease of renal macrophage content, by CD68 IHC (data not shown) or by mRNA expression (**Supplementary Table S1**). We then sought to determine whether there was a shift in the balance in M1 versus M2 macrophage lineages. No net change in renal macrophage subtypes were detected as both M1 (IL-12 and TNF-α), as well as M2 (TGF-β and CD206) cytokine mRNAs were similarly induced by DOX and unchanged in the CD40 ASO-treated animals (**Supplementary Table S1**).

### CD40 expression, but not CD40L, is enriched in renal glomeruli and elevated in DOX nephropathy

**Figure 5:**
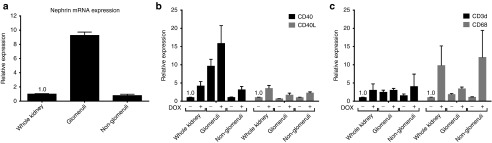
**CD40, but not CD40L, is enriched in glomeruli and its glomerular expression is increased in DOX nephropathy**. Glomerular and nonglomerular fractions were separated from collagenase-digested kidneys by cardiac injection of magnetic beads prior to organ harvest followed by positive selection of glomeruli using a magnet and negative selection of nonglomeruli collected as the flow-thru. (**a**) The glomerular-specific marker nephrin measured by quantitative real-time polymerase chain reaction (qRT-PCR) in whole kidney homogenates and the kidney fractions. Kidney fractions were prepared from healthy or DOX nephropathy mice and (**b**) CD40 and CD40L or (**c**) CD3d and CD68 qRT-PCR were performed in whole kidney homogenates and the kidney fractions. Data are normalized to whole kidney (−) DOX treatment = 1.0. Mean ± SEM, *n* = 4/group.

Previous work had suggested increased CD40 expression in a variety of cell types within the renal cortex following doxorubicin administration. Specifically, early in DOX nephropathy, CD40 appeared elevated on glomerular cells and then becomes more widespread throughout the renal cortex. We evaluated the renal suborgan expression of CD40 mRNA in mice with early stage doxorubicin-induced (DOX) nephropathy and healthy controls. To isolate glomeruli, animals were perfused with a suspension of magnetic beads, kidneys were then mechanically and enzymatically digested and glomeruli were purified from the resulting tissue digest using a magnet. A 10-fold enrichment of the glomerular-specific marker, nephrin, was achieved in glomerular fractions (**Figure 5a**). Two weeks following DOX administration, whole kidney CD40 and CD40L mRNA expression were increased approximately fourfold (**Figure 5b**). In both healthy and DOX-injured kidneys, CD40 expression was enriched in glomerular fractions. In contrast, CD40L expression was similar within all renal fractions in either healthy or DOX-injured kidneys (**Figure 5b**). Markers of T cells (CD3d mRNA) and macrophages (CD68 mRNA) were modestly elevated in glomerular fractions; however following DOX injury, large increases in CD68 were also observed in nonglomerular fractions (**Figure 5c**).

### CD40-dependent inflammation was enhanced in DOX nephropathy and attenuated by CD40 ASO treatment

To determine if DOX nephropathy sensitized kidneys to CD40 signaling and whether CD40 ASO treatment could attenuate this effect, the activating CD40 mAb was given to mice with either low or high DOX nephropathy that had also received control or CD40 ASO treatments. As previously described, DOX nephropathy was initiated with a single IV dose of 10.5 mg/kg DOX, but unlike the previous experiment, the low and high DOX injury outliers were studied. At 29, 33, and 36 days following DOX administration, 25 mg/kg control or CD40 ASO treatments were given followed by 25 μg of the CD40 mAb on day 39. Kidneys were harvested 24 hours later.

**Figure 6:**
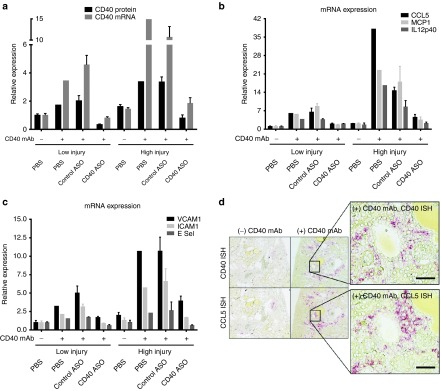
**Kidney CD40-dependent inflammation, increased in DOX nephropathy, was attenuated by CD40 antisense oligonucleotide (ASO) treatments**. Following 10.5 mg/kg DOX, mice were divided into those with exhibiting low or high DOX injury based on body weight change and proteinuria. Three 25 mg/kg ASO treatments were then given 29, 33, and 36 days following the DOX administration. Next, the activating CD40 mAb was given on day 39 and kidneys were harvested on day 40. (**a**) CD40 protein and mRNA measurements (**b** and **c**) and CD40-dependent inflammation were measured from whole kidney quantitative real-time polymerase chain reaction analyses. (**d**) CD40 and CCL5 *in situ* hybridization in kidneys from high DOX injury mice in the absence or presence of the CD40 mAb. Data are normalized to low injury (−) CD40 mAb phosphate buffered saline (PBS) = 1.0. Mean ± SEM, *n* = 1–3/group, scale bar = 50 μm.

In PBS-treated mice receiving the CD40 mAb, there were larger increases in kidney CD40 protein and mRNA in the high DOX injury mice relative to low DOX injury mice (**Figure 6a**). High DOX injury mice also had greater kidney inflammatory responses to the CD40 mAb, demonstrated by an approximately 40- versus 7-fold CCL5 increase in the high and low PBS-treated mice, respectively (**Figure 6b**). CD40 ASO treatment sharply attenuated or prevented CD40 mAb-dependent inflammatory responses by up to 90%.

Analysis of the distribution of CD40 and CCL5 mRNA expression by ISH revealed a focal distribution pattern in the kidney cortex in DOX nephropathy similar to that observed in healthy mice receiving the activating CD40 mAb. Analysis of successive sections revealed similar, but possibly not identical, sites expressing CD40 and CCL5 within the cortex (**Figure 6d**).

### CD40-dependent inflammation was enhanced in obstructed kidneys and attenuated by CD40 ASO treatment

Unilateral ureter obstruction (UUO) studies were performed to determine the efficacy of CD40 ASO treatment to blunt CD40-dependent inflammation in a renal disease model not driven by glomerular injury. Additionally, since ASOs have been shown to target cortical interstitial cells, ASO treatments given when glomerular filtration rate is restricted should still result in an attenuation of CD40-dependent cortical inflammatory responses. CD40 ASO treatments were given to mice 1, 4, and/or 8 days following UUO using three different dosing paradigms: three administrations of 25 mg/kg (treatments on day 1, 4, and 8; 75 mg/kg total dose) or two administrations of 25 mg/kg (treatments on day 4 and 8; 50 mg/kg total dose) or a single administration of 25 mg/kg (treatment on day 8). Mice were given the CD40 mAb on day 10 and then kidneys were harvested on day 11.

**Figure 7:**
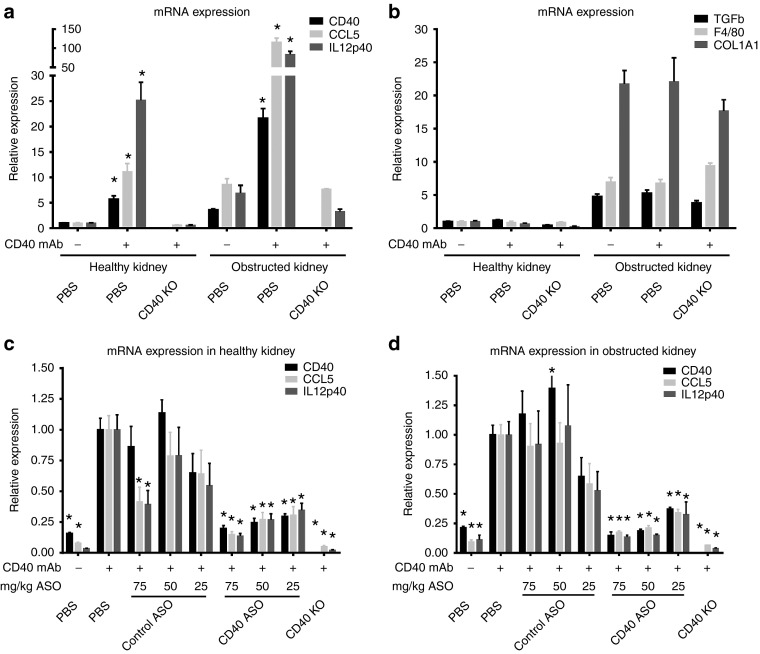
**Ureter obstruction amplified CD40-dependent inflammation and these changes were attenuated by CD40 antisense oligonucleotide (ASO) treatments**. Unilateral ureter obstruction (UUO) was performed and 10 days afterward mice received the activating CD40 mAb and then kidneys were harvested 24 hours later. (**a**) Inflammatory marker (CD40, CCL5, and IL12p40) and (**b**) fibrosis or macrophage marker (TGFβ, F4/80, and COL1A1) mRNAs were measured by quantitative real-time polymerase chain reaction (qRT-PCR) and compared between the healthy contralateral kidney and obstructed kidney from control mice or wild-type and CD40 knockout mice receiving the activating CD40 mAb. To evaluate the efficacy of CD40 ASO treatment to blunt CD40-dependent inflammation during UUO, control, or CD40 ASO treatments or CD40 knockout mice were subjected to UUO and then given the activating CD40 mAb. Three different ASO dosing regimens were used such that cohorts of mice received a combined ASO dose of 75, 50, or 25 mg/kg. In all cohorts, the activating CD40 mAb was given 2 days after the last ASO dose and then kidneys were harvested 24 hours afterward. CD40, CCL5, and IL12p40 mRNA levels were measured by qRT-PCR in the (**c**) healthy and (**d**) contralateral obstructed kidney. Data are normalized to the healthy kidney (−) CD40 mAb phosphate buffered saline (PBS) = 1.0 for panels **a and b** and the (+) CD40 mAb PBS = 1.0 for panels **c and d**. Mean ± SEM, *n* = 4/group, *P < 0.05 versus (+) PBS group.

**Figure 8:**
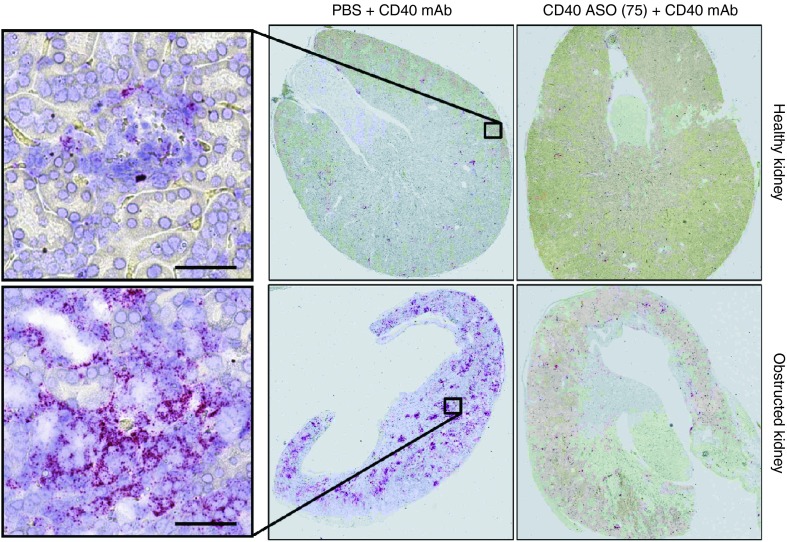
**Interstitial CD40 expression was primed for activation in kidneys with ureter obstruction and could be markedly reduced with CD40 antisense oligonucleotide (ASO) treatment**. Kidney CD40 *in situ* hybridization performed in the healthy and obstructed kidneys in UUO mice receiving phosphate buffered saline (PBS) + CD40 mAb or 75 mg/kg CD40 ASO + CD40 mAb. Scale bar = 50 μm.

CD40-dependent inflammation was amplified in the obstructed kidney following CD40 Ab administration (**Figure 7a**). For example, CD40 mAb treatment increased renal CD40, CCL5 and IL-12 by 22-, 116- and 84-fold, respectively, in the obstructed kidney, whereas in the healthy contralateral kidney, CD40, CCL5, and IL12p40 were increased 4–10-fold less. Ureter obstruction resulted in a large increase in renal fibrosis and macrophage infiltration markers (TGFβ1, COL1A1, and F4/80), which were unaffected by either CD40 activation or in CD40 knockout mice (**Figure 7b**). CD40 ASO treatments mitigated CD40 Ab-dependent induction of inflammation in a dose-dependent manner in both the healthy contralateral and obstructed kidneys (**Figure 7c**,d). The efficacy of the CD40 ASO treatment in the obstructed kidney was noteworthy given its heightened response to the CD40 Ab. In the obstructed kidneys from either the 50 or 75 mg/kg total dose CD40 ASO groups, CD40, CCL5, and IL12p40 were reduced by 80–85%, which approached that observed in the CD40 knockout mice (**Figure 7d**). Large increases in cortical interstitial CD40 mRNA expression were observed in the obstructed kidneys following CD40 mAb administration, which were largely eliminated in mice receiving CD40 ASO treatments (**Figure 8**).

## Discussion

The experiments described herein were conducted to (i) identify renal sites susceptible to CD40 activation, (ii) determine whether antisense inhibition of CD40 could ameliorate disease in a mouse model of glomerular injury, (iii) test the hypothesis that CD40 is primed in kidneys with preexisting injury, and (iv) determine if CD40-dependent renal inflammation could be abrogated with CD40 ASO treatment. Our results demonstrate that CD40 ASO treatment resulted in a 75–90% reduction of renal CD40 across several models of kidney injury. CD40 ASO treatment mitigated functional, transcriptional, and histologic assessments of DOX nephropathy. Moreover, these studies demonstrate that CD40 activation, which is primed at renal cortical sites following kidney injury, results in a myriad of inflammatory responses which can be effectively attenuated with CD40 ASO treatment.

DOX nephropathy models the progressive podocyte depletion, tubulointerstitial inflammation, and the development of glomerulosclerosis similar to the pathogenic changes observed in FSGS.^26^ Previous studies have demonstrated that blockade of CD40-CD40L signaling via administration of a CD40L antagonistic antibody was effective at mitigating disease.^20,27^ Our data further support an important role of CD40 in a mouse model of chronic kidney disease and also suggest that renal expression of CD40 has an important role to promote inflammation and disease. Studies have demonstrated enhanced renal CD40 in renal biopsy samples following allograft rejection, recurrent FSGS, and in lupus nephritis.^13,14,21^ Collectively, these data suggest therapeutic inhibition of renal CD40 could provide benefit in patients with kidney inflammation or glomerulonephritis.

To our knowledge, this is the first demonstration that CD40 activation results in an induction of a diverse set of inflammatory mediators such as adhesion molecules, cytokines, and chemokines within the kidney and that these responses are amplified with pre-existing injury. The pattern of CD40 induction within the cortical interstitium was similar in healthy, DOX injured, and obstructed kidneys, although the intensity of CD40 expression was highest in the obstructed kidney. Previous studies have suggested a role of CD40 signaling in the cortical inflammatory responses to DOX-induced injury.^27^ Additionally, studies have shown that that renal tissue exhibits high basal CD40 vascular expression that is subject to a greater level of induction following LPS compared to vascular CD40 expressed in the heart, lungs, small intestine, and brain.^28^ We also observed that basal CD40 expression was more abundant in the kidney compared to the colon, liver, or heart, and can be increased by up to 20-fold following CD40 activation.

The putative sites of renal CD40 activation may be glomerular cells, interstitial fibroblasts, endothelial cells, dendritic cells, macrophages, or other immune cell infiltrates. Previous work demonstrated strong upregulation of CD40 in glomerular cells 2 weeks after DOX nephropathy induction followed by loss of glomerular expression and increased expression in proximal tubular epithelium (PTEC) and cortical interstitium.^20,27^ Histological studies have demonstrated a rich network of fibroblasts and dendritic cells within the renal interstitium^29,30,31^ that would be predicted to be highly responsive to CD40 activation. *In vitro* studies have demonstrated that endothelial responses to CD40 activation results in a broad and profound stress response resulting in activation of diverse inflammatory and thrombotic pathways.^32^

Despite this wealth of knowledge of CD40 expression, the contribution of these cell types to CD40 signaling that underlie renal disease is still unclear. Our data demonstrates low basal CD40 expression within glomeruli and tubular epithelial cells which during disease states becomes highly enriched within glomeruli and cortical interstitial sites. Interestingly, the particular disease state (*e.g.*, DOX nephropathy or UUO) did not make for qualitative differences in the distribution of renal CD40 expression, nor did the administration of the activating CD40 mAb. Importantly, we show that activation of renal CD40 using the CD40 mAb is mostly dependent on a myeloid-derived cell population, as bone marrow CD40 deficiency results in almost complete attenuation of CD40-depedent renal inflammation. These data do not illuminate the precise cell type mediating these effects, but do suggest a kidney-resident, but myeloid-derived, cell as the CD40 mAb did not result in increased renal macrophages, T cells or B cells. Future studies are needed to determine the relative contributions of myeloid (*e.g.*, CD11c+ DCs and CD11b+ macrophages) and non-myeloid cell-specific CD40 activity in the context of renal inflammation and disease. Additionally, studies are also needed to clarify the dependency of CD40L in the context of CD40 inhibition, as reports have established non-canonical pathways of CD40L signaling that do not involve CD40 (refs. 33,34,35,36). Finally, studies to precisely determine the relative potency of ASOs across unique cell populations that reside in the renal cortex during disease will be an important advance in defining the therapeutic potential of the Generation 2.5 chemistry platform for chronic kidney disease.

The robust potency of CD40 ASO to mitigate CD40 Ab-induced renal inflammation was striking. Although it is well established that the renal cortex, specifically the PTEC, is the site with the greatest concentration of ASO, few studies have investigated the distribution and activity of ASOs at non-PTEC sites within the renal cortex.^37^ Additionally, the activity of Generation 2.5 ASOs, which represent the most potent and advanced class of ASOs,^38^ have only begun to be characterized in the kidney. In a recent report, Hung *et al*.^25^ demonstrated 93% target inhibition in the kidney with a cET ASO targeting Malat1, which was improved compared to the 72% target inhibition observed with the generation 2.0 MOE gapmer Malat1 ASO. Here we demonstrate that cells, myeloid-derived and possibly endothelial cells, in the renal cortex, distinct from the PTECs, can be effectively targeted with Generation 2.5 ASOs.

Experiments in the UUO model support the concept that CD40 is primed and can be activated within the interstitium, but not the glomerulus or tubular epithelium, as the ASO treatments and the CD40 mAb were given after ureter obstruction when there is severely restricted or absent glomerular filtration rate.^39^ For example, a single dose of the CD40 ASO at 25 mg/kg given 8 days after ureter obstruction resulted in an attenuation of CD40 and the CD40-dependent inflammatory markers CCL5 and IL12p40 (**Figure 7d**). These data suggest that renal cortical ASO distribution is not primarily dependent upon glomerular filtration and reabsorption processes, but can occur via a tubular-independent renovascular ASO distribution pathway. Such data raises the prospect that in patients with reduced glomerular filtration rate might still be benefit from CD40 ASO therapy.

In summary, antisense inhibition of CD40 in established DOX nephropathy mitigated functional, transcriptional, and pathological assessments of disease severity and sharply attenuated CD40-dependent inflammatory responses in the kidney. The ability of CD40 ASO treatment to sharply attenuate or prevent CD40-dependent renal inflammation in multiple models of kidney injury highlights the potential of antisense therapy to treat such CD40-dependent inflammatory states. This could prove beneficial in patients with aberrant or excessive renal CD40 activation. The source of such activation could be similar to that modeled in these studies with the CD40 activating Ab, as would be the case in patients with high titers of autoantibodies to CD40 (ref. 21). Additionally, activated T cells and platelets are a rich source of CD40L and sCD40L, respectively, which could act as triggers for CD40-dependent renal inflammation. Identifying patients with such aberrant CD40 signaling would be important in selecting patient subpopulations that could maximally benefit from a CD40 ASO therapeutic.

## Materials and methods

*CD40 ASO lead determination.* Antisense phosphorothioate oligonucleotides (ASOs) with 5'-methyl cytosine and containing 2'-O-methoxyethyl (MOE, e) and 2-O, 4-C-((S)-ethylidene)-D-ribose (cEt, k) modified sugars were synthesized at Isis Pharmaceuticals (Carlsbad, CA) as described previously.^40^ Chimeric ASOs containing both Generation 2.0 (*i.e.*, MOE) and Generation 2.5 (*i.e.*, cET) chemistries 16 bases in length had the modified sugar pattern, eek-10-kke, wherein the three terminal 5' and 3' nucleotides contain modified sugars that flank 10 nucleotides with unmodified sugars were screened for activity and tolerability. Extensive *in vitro* activity screens were performed followed by *in vivo* tolerability and activity studies to identify a lead CD40 ASO. Additional SAR was performed around this sequence to identify an active and well tolerated lead (CD40 ASO) containing a kkk-10-kkk sugar composition and with nucleotide sequence CAGATTTATTTAGCCA. A control ASO of the same chemical class as CD40 ASO, but not hybridizing to any known murine RNA sequences, was used and had the following nucleotide sequence, GGCCAATACGCCGTCA. The Malat1 (NCBI Accession NR_002847) ASO with kkk-10-kkk sugar composition had the following nucleotide sequence, CTAGTTCACTGAATGC.

*In vitro CD40 ASO activity.* *In vitro* CD40 activity of ASOs was tested in two screens using thioglycollate-elicited peritoneal macrophage (TG-MΦ) treated with 10 μmol/l ASO for 1 hour in media (10% fetal bovine serum, 1× antibiotic/antimycotic Dulbecco's Modified Eagle's Medium), washed with PBS, and the media replaced before activating the cells (**Supplementary Figure S1**). In the first screen, 0.5 μg/ml LPS was added and the cells were harvested at 4, 12, and 24 hours. In the second screen, CD40 was activated by 100 ng/ml IFN-γ (R&D Systems, Minneapolis, MN) for 4 hours followed by 10 μg/ml activating anti-CD40 monoclonal antibody (mAb), clone 3/23 (Abcam, Cambridge, MA) for 4, 15, or 24 hours. Media was collected to measure CCL5 protein levels and the cells were lysed for mRNA expression analysis.

*Animal studies.* All animal procedures were reviewed and approved by the Isis Institutional Animal Care and Use Committee and conducted in conformity with the Public Health Service Policy on Humane Care and Use of Laboratory Animals. All mice were housed in a pathogen-free environment and given food and water *ad libitum*. Male wild-type C57BL/6 (WT) (JAX #000664) and CD40 knockout (CD40 KO) (JAX #002928) mice were used for studies not utilizing DOX. Male BALB/c mice (Charles River, San Diego, CA) were used for DOX studies. CD40 and a control ASO were SC injected once weekly unless otherwise specified at doses ranging from 5 to 100 mg/kg. ASO dosing solutions were prepared in sterile PBS and given at a dose volume of 10 ml/kg body weight.

*In vivo* tolerability of the lead CD40 ASO was assessed in male inflammation-naive C57BL/6 mice after 4 weeks of SC administration of 50 and 100 mg/kg/week for a combined ASO dose of 200 or 400 mg/kg, respectively. As shown in **Supplementary Figure S2**, body weight changes, plasma total bilirubin and BUN were unchanged in mice receiving up to 100 mg/kg/week. At 100 mg/kg/week, there were perturbations in spleen size and plasma ALT, which were not observed in the 50 mg/kg/week dose group. Additionally, there were no improvements in activity with dosing beyond 50 mg/kg/week. Therefore, our renal pharmacology studies were performed using doses less than 50 mg/kg/week.

LPS (055:B5, Sigma) and the activating CD40 mAb (clone 3/23, Abcam) were administered IP and IV, respectively. Anesthetized mice were euthanized by cervical dislocation and blood was collected by cardiac puncture and kidneys harvested and cut in half, in the transverse plane, for mRNA expression analysis, protein analysis, and histology.

*Bone marrow transplantation.* Male WT and CD40 KO mice were irradiated with 7 Gy and then given IV ~400,000 BM donor cells. Six weeks following the bone marrow transplantation, reconstitution was confirmed by measurement of CD40 mRNA expression in peripheral blood leukocyte population (**Supplementary Figure S4**).

*Glomerular isolation.* Isolation of glomeruli was performed by the method described by Takemoto *et al*.^41^ Briefly, magnetic 4.5 μm Dynabeads (ThermoFisher, Waltham, MA) were perfused at animal sacrifice via the left ventricle. Kidneys were harvested and digested in a collagenase- (Worthington Biochemical Corporation, Lakewood, NJ), hyaluronidase-(Sigma-Aldrich, St. Louis, MO) and DNaseI-(ThermoFisher) containing buffer. After mechanical disruption, tissue was centrifuged and the pellet was washed. Glomeruli containing Dynabeads were collected by a magnetic particle concentrator.

*Renal function assessment.* An estimate of renal function was determined by measuring the elimination of plasma FITC-Inulin using a modified version of the FITC-Inulin clearance method.^42^ Briefly, 8 μl/g body weight of 2.5% FITC-Inulin was given IV and blood was collected 2 hours afterward. Proteinuria was assessed by determining the ratio of urinary albumin and creatinine (ELISA, Kamiya Biomedical , Seattle, WA and Olympus AU400 Clinical Analyzer respectively).

*RNA purification and quantitative reverse transcription polymerase chain reaction (qRT-PCR).* TG-MΦ or tissues were homogenized and RNA prepared using the PureLink Pro 96 RNA kit (ThermoFisher). mRNA levels were measured using the One-Step RT-PCR kit, gene-specific TaqMan probe and primer sets and the StepOne Plus (ThermoFisher). Sequence information for probe and primer sets can be found in **Supplementary Table S2**. mRNA levels for target genes were normalized to either total RNA (Ribogreen) or GAPDH mRNA expression.

*ELISA.* ELISAs were performed for CCL5 (eBioscience, San Diego, CA and R&D Systems), CD40 (R&D Systems), and urinary albumin (Kamiya Biomedical) according to the manufacturer's instructions. Kidney homogenates were prepared for ELISA by homogenization in RIPA lysis buffer (ThermoFisher) containing a protease inhibitor cocktail (complete, Roche, Indianapolis, IN).

*Histology.* Kidneys were fixed in 10% neutral buffered formalin, dehydrated in graded ethanol and embedded in paraffin, sectioned and stained using hematoxylin and eosin or periodic acid-Schiff. IHC was performed using antibodies against CD3d (Serotec, Raleigh, NC) and CD68 (Boster Biologics, Pleasanton, CA) and ASO (generated in-house). CD40, CCL5, or Malat1 mRNA expression was detected via ISH using the QuantiGene ViewRNA tissue assay (Affymetrix Santa Clara, CA) according to the manufacturer's instructions and probes. No probe controls or tissue from CD40 knockout mice were used for negative controls.

*Statistics.* Data are reported as means ± SEM. Two-tailed Student's *t*-test was used for comparisons between two groups. One-way analysis of variance and the Holm-Sidak *post hoc* test (SigmaStat, Systat Software San Jose, CA) used to determine significance relative to the PBS vehicle control for studies with more than two groups. Statistical significance was considered when *P* < 0.05.

**SUPPLEMENTARY MATERIAL** **Figure S1.** Malat1 ASO activity (ISH) and distribution (IHC) in the kidney. **Figure S2.** Tolerability and activity evaluation of the lead CD40 ASO in inflammation-naïve mice. **Figure S3.** Dose responsive reduction of CD40-dependent inflammation with CD40 ASO treatments and leukocyte mRNA marker expression following CD40 mAb administration. **Figure S4.** CD40 mRNA expression in blood PBMC cells 6 weeks post BMT. **Table S1.** CD40 ASO treatment effects on kidney T cell and macrophage markers in DOX nephropathy. **Table S2.** Primer and probe sequences used for qRT-PCR.

## References

[bib1] Coresh, J, Selvin, E, Stevens, LA, Manzi, J, Kusek, JW, Eggers, P et al. (2007). Prevalence of chronic kidney disease in the United States. JAMA 298: 2038–2047. 17986697 10.1001/jama.298.17.2038

[bib2] Moll, S, Meier, M, Formentini, I, Pomposiello, S and Prunotto, M (2014). New renal drug development to face chronic renal disease. Expert Opin Drug Discov 9: 1471–1485. 25195802 10.1517/17460441.2014.956075

[bib3] Bohle, A, Mackensen-Haen, S, von Gise, H, Grund, KE, Wehrmann, M, Batz, C et al. (1990). The consequences of tubulo-interstitial changes for renal function in glomerulopathies. A morphometric and cytological analysis. Pathol Res Pract 186: 135–144. 2315207 10.1016/S0344-0338(11)81021-6

[bib4] Grewal, IS and Flavell, RA (1997). The CD40 ligand. At the center of the immune universe? Immunol Res 16: 59–70. 9048208 10.1007/BF02786323

[bib5] Kato, K, Santana-Sahagún, E, Rassenti, LZ, Weisman, MH, Tamura, N, Kobayashi, S et al. (1999). The soluble CD40 ligand sCD154 in systemic lupus erythematosus. J Clin Invest 104: 947–955. 10510335 10.1172/JCI7014 PMC408556

[bib6] Mach, F (1998). Reduction of atherosclerosis in mice by inhibition of CD40 signalling. Nature 394: 200–203. 9671306 10.1038/28204

[bib7] Heeschen, C, Dimmeler, S, Hamm, CW, van den Brand, MJ, Boersma, E, Zeiher, AM et al.; CAPTURE Study Investigators. (2003). Soluble CD40 ligand in acute coronary syndromes. N Engl J Med 348: 1104–1111. 12646667 10.1056/NEJMoa022600

[bib8] Criswell, LA (2010). Gene discovery in rheumatoid arthritis highlights the CD40/NF-kappaB signaling pathway in disease pathogenesis. Immunol Rev 233: 55–61. 20192992 10.1111/j.0105-2896.2009.00862.x

[bib9] Suttles, J and Stout, RD (2009). Macrophage CD40 signaling: a pivotal regulator of disease protection and pathogenesis. Semin Immunol 21: 257–264. 19540774 10.1016/j.smim.2009.05.011

[bib10] Danese, S, Sans, M and Fiocchi, C (2004). The CD40/CD40L costimulatory pathway in inflammatory bowel disease. Gut 53: 1035–1043. 15194658 10.1136/gut.2003.026278 PMC1774101

[bib11] Ruth, AJ, Kitching, AR, Semple, TJ, Tipping, PG and Holdsworth, SR (2003). Intrinsic renal cell expression of CD40 directs Th1 effectors inducing experimental crescentic glomerulonephritis. J Am Soc Nephrol 14: 2813–2822. 14569091 10.1097/01.asn.0000091381.60059.fb

[bib12] van Kooten, C, Gerritsma, JS, Paape, ME, van Es, LA, Banchereau, J and Daha, MR (1997). Possible role for CD40-CD40L in the regulation of interstitial infiltration in the kidney. Kidney Int 51: 711–721. 9067903 10.1038/ki.1997.102

[bib13] Woltman, AM, de Haij, S, Boonstra, JG, Gobin, SJ, Daha, MR and van Kooten, C (2000). Interleukin-17 and CD40-ligand synergistically enhance cytokine and chemokine production by renal epithelial cells. J Am Soc Nephrol 11: 2044–2055. 11053480 10.1681/ASN.V11112044

[bib14] Yellin, MJ, D'Agati, V, Parkinson, G, Han, AS, Szema, A, Baum, D et al. (1997). Immunohistologic analysis of renal CD40 and CD40L expression in lupus nephritis and other glomerulonephritides. Arthritis Rheum 40: 124–134. 9008608 10.1002/art.1780400117

[bib15] Biancone, L, Andres, G, Ahn, H, DeMartino, C and Stamenkovic, I (1995). Inhibition of the CD40-CD40ligand pathway prevents murine membranous glomerulonephritis. Kidney Int 48: 458–468. 7564113 10.1038/ki.1995.314

[bib16] Early, GS, Zhao, W and Burns, CM (1996). Anti-CD40 ligand antibody treatment prevents the development of lupus-like nephritis in a subset of New Zealand black x New Zealand white mice. Response correlates with the absence of an anti-antibody response. J Immunol 157: 3159–3164. 8816428

[bib17] Kalled, SL, Cutler, AH, Datta, SK and Thomas, DW (1998). Anti-CD40 ligand antibody treatment of SNF1 mice with established nephritis: preservation of kidney function. J Immunol 160: 2158–2165. 9498753

[bib18] Ripoll, È, Merino, A, Herrero-Fresneda, I, Aran, JM, Goma, M, Bolaños, N et al. (2013). CD40 gene silencing reduces the progression of experimental lupus nephritis modulating local milieu and systemic mechanisms. PLoS One 8: e65068. 23799000 10.1371/journal.pone.0065068 PMC3683035

[bib19] Ripoll, E, Pluvinet, R, Torras, J, Olivar, R, Vidal, A, Franquesa, M et al. (2011). *In vivo* therapeutic efficacy of intra-renal CD40 silencing in a model of humoral acute rejection. Gene Ther 18: 945–952. 21472009 10.1038/gt.2011.39

[bib20] Kairaitis, L, Wang, Y, Zheng, L, Tay, YC, Wang, Y and Harris, DC (2003). Blockade of CD40-CD40 ligand protects against renal injury in chronic proteinuric renal disease. Kidney Int 64: 1265–1272. 12969144 10.1046/j.1523-1755.2003.00223.x

[bib21] Delville, M, Sigdel, TK, Wei, C, Li, J, Hsieh, SC, Fornoni, A et al. (2014). A circulating antibody panel for pretransplant prediction of FSGS recurrence after kidney transplantation. Sci Transl Med 6: 256ra136. 10.1126/scitranslmed.3008538 PMC4394366 25273097

[bib22] Kirk, AD (2009). 4D11: The Second Mouse? Am J Transplant 9: 1701–1702. 19660019 10.1111/j.1600-6143.2009.02749.x

[bib23] Sidiropoulos, PI and Boumpas, DT (2004). Lessons learned from anti-CD40L treatment in systemic lupus erythematosus patients. Lupus 13: 391–397. 15230298 10.1191/0961203304lu1032oa

[bib24] Okimura, K, Maeta, K, Kobayashi, N, Goto, M, Kano, N, Ishihara, T et al. (2014). Characterization of ASKP1240, a fully human antibody targeting human CD40 with potent immunosuppressive effects. Am J Transplant 14: 1290–1299. 24731050 10.1111/ajt.12678 PMC4225473

[bib25] Hung, G, Xiao, X, Peralta, R, Bhattacharjee, G, Murray, S, Norris, D et al. (2013). Characterization of target mRNA reduction through in situ RNA hybridization in multiple organ systems following systemic antisense treatment in animals. Nucleic Acid Ther 23: 369–378. 24161045 10.1089/nat.2013.0443

[bib26] Wang, Y, Wang, YP, Tay, YC and Harris, DC (2000). Progressive adriamycin nephropathy in mice: sequence of histologic and immunohistochemical events. Kidney Int 58: 1797–1804. 11012915 10.1046/j.1523-1755.2000.00342.x

[bib27] Lee, VW, Qin, X, Wang, Y, Zheng, G, Wang, Y, Wang, Y et al. (2010). The CD40-CD154 co-stimulation pathway mediates innate immune injury in adriamycin nephrosis. Nephrol Dial Transplant 25: 717–730. 19889873 10.1093/ndt/gfp569

[bib28] Vowinkel, T, Wood, KC, Stokes, KY, Russell, J, Krieglstein, CF and Granger, DN (2006). Differential expression and regulation of murine CD40 in regional vascular beds. Am J Physiol Heart Circ Physiol 290: H631–H639. 16172156 10.1152/ajpheart.00733.2005

[bib29] Kaissling, B and Le Hir, M (2008). The renal cortical interstitium: morphological and functional aspects. Histochem Cell Biol 130: 247–262. 18575881 10.1007/s00418-008-0452-5 PMC2491705

[bib30] Kitching, AR (2014). Dendritic cells in progressive renal disease: some answers, many questions. Nephrol Dial Transplant 29: 2185–2193. 24739483 10.1093/ndt/gfu076

[bib31] Snelgrove, SL, Kausman, JY, Lo, C, Lo, C, Ooi, JD, Coates, PT et al. (2012). Renal dendritic cells adopt a pro-inflammatory phenotype in obstructive uropathy to activate T cells but do not directly contribute to fibrosis. Am J Pathol 180: 91–103. 22079432 10.1016/j.ajpath.2011.09.039

[bib32] Pluvinet, R, Olivar, R, Krupinski, J, Herrero-Fresneda, I, Luque, A, Torras, J et al. (2008). CD40: an upstream master switch for endothelial cell activation uncovered by RNAi-coupled transcriptional profiling. Blood 112: 3624–3637. 18669876 10.1182/blood-2008-03-143305

[bib33] El Fakhry, Y, Alturaihi, H, Yacoub, D, Liu, L, Guo, W, Leveillé, C et al. (2012). Functional interaction of CD154 protein with α5β1 integrin is totally independent from its binding to αIIbβ3 integrin and CD40 molecules. J Biol Chem 287: 18055–18066. 22461623 10.1074/jbc.M111.333989 PMC3365732

[bib34] Okwor, I, Jia, P and Uzonna, JE (2015). Interaction of Macrophage Antigen 1 and CD40 Ligand Leads to IL-12 Production and Resistance in CD40-Deficient Mice Infected with Leishmania major. J Immunol 195: 3218–3226. 26304989 10.4049/jimmunol.1500922

[bib35] Wolf, D, Hohmann, JD, Wiedemann, A, Bledzka, K, Blankenbach, H, Marchini, T et al. (2011). Binding of CD40L to Mac-1's I-domain involves the EQLKKSKTL motif and mediates leukocyte recruitment and atherosclerosis–but does not affect immunity and thrombosis in mice. Circ Res 109: 1269–1279. 21998326 10.1161/CIRCRESAHA.111.247684 PMC3291815

[bib36] Nathan, MJ, Mold, JE, Wood, SC, Csencsits, K, Lu, G, Eichwald, EJ et al. (2004). Requirement for donor and recipient CD40 expression in cardiac allograft rejection: induction of Th1 responses and influence of donor-derived dendritic cells. J Immunol 172: 6626–6633. 15153477 10.4049/jimmunol.172.11.6626

[bib37] Geary, RS (2009). Antisense oligonucleotide pharmacokinetics and metabolism. Expert Opin Drug Metab Toxicol 5: 381–391. 19379126 10.1517/17425250902877680

[bib38] Seth, PP, Siwkowski, A, Allerson, CR, Vasquez, G, Lee, S, Prakash, TP et al. (2009). Short antisense oligonucleotides with novel 2'-4' conformationaly restricted nucleoside analogues show improved potency without increased toxicity in animals. J Med Chem 52: 10–13. 19086780 10.1021/jm801294h

[bib39] Harris, RH and Gill, JM (1981). Changes in glomerular filtration rate during complete ureteral obstruction in rats. Kidney Int 19: 603–608. 7241893 10.1038/ki.1981.58

[bib40] Seth, PP, Siwkowski, A, Allerson, CR, Vasquez, G, Lee, S, Prakash, TP et al. (2008). Design, synthesis and evaluation of constrained methoxyethyl (cMOE) and constrained ethyl (cEt) nucleoside analogs. Nucleic Acids Symposium Series 52: 553–554. 18776499 10.1093/nass/nrn280

[bib41] Takemoto, M, Asker, N, Gerhardt, H, Lundkvist, A, Johansson, BR, Saito, Y et al. (2002). A new method for large scale isolation of kidney glomeruli from mice. Am J Pathol 161: 799–805. 12213707 10.1016/S0002-9440(10)64239-3 PMC1867262

[bib42] Qi, Z, Whitt, I, Mehta, A, Jin, J, Zhao, M, Harris, RC et al. (2004). Serial determination of glomerular filtration rate in conscious mice using FITC-inulin clearance. Am J Physiol Renal Physiol 286: F590–F596. 14600035 10.1152/ajprenal.00324.2003

