# Supplementary material for: CD40 Generation 2.5 Antisense Oligonucleotide Treatment Attenuates Doxorubicin-induced Nephropathy and Kidney Inflammation
Source: Mol Ther Nucleic Acids. 2015 Dec 1;4(12):e265–. doi: 10.1038/mtna.2015.40 (PMC5014534; doi:10.1038/mtna.2015.40)
Supplement: Supplementary Material [file mtna201540x1.pdf]

Supplementary Material

Figure S1. Malat1 ASO activity (ISH) and distribution (IHC) in the kidney. Mice were treated with PBS or 100 mg/kg Malat1 ASO for 4 wks and kidney Malat1 RNA expression was evaluated in (A) PBS-treated mice or (B) Malat1 ASO-treated mice by ISH. (C) The distribution of ASO accumulation by IHC using an anti-ASO Ab. Black arrows denote interstitial sites demonstrating Malat1 ASO activity (B) and accumulation (C). Scale bar = 50 μm

Figure S2. Tolerability and activity evaluation of the lead CD40 ASO in inflammation-naïve mice. The lead CD40 ASO was evaluated in C57BL/6 mice after four weeks of SC administration of 50 and 100 mg/kg/wk. Basic measures of tolerability (body and spleen weights, plasma bilirubin, BUN and ALT) were assessed and CD40 RT-PCR was performed in the liver and kidney.

Figure S3. Dose responsive reduction of CD40-dependent inflammation with CD40 ASO treatments and leukocyte mRNA marker expression following CD40 mAb administration. (A) To evaluate dose-responsive CD40 ASO activity following CD40-dependent inflammation, C57BL/6 mice were given 40, 15 or 5 mg/kg ASO treatments on days 0 and 7, followed by 25 μg of the activating CD40 mAb on day 10, kidneys were harvested on day 11 and CD40, CCL5 and IL12p40 mRNA levels were measured by RT-PCR. (B) mRNA markers of specific leukocyte populations (F4/80, CD19 and CD3d) were analyzed by RT-PCR in control mice and mice administered the CD40 mAb 24 hrs prior to sacrifice.

Figure S4. CD40 mRNA expression in blood PBMC cells 6 weeks post BMT. Verification of bone marrow reconstitution was performed by RT-PCR using two different CD40 primer probe sets that either amplify CD40 mRNA in WT and CD40 KO leukocytes (PPSet #3483) or only in WT

leukocytes (PPSet #1280). The six groups shown were treated with CD40 ASO and/or CD40 Ab as described in Figure 3f.

Table S1. CD40 ASO treatment effects on kidney T cell and macrophage markers in DOX nephropathy.

Table S2. Primer and probe sequences used for RT-PCR.

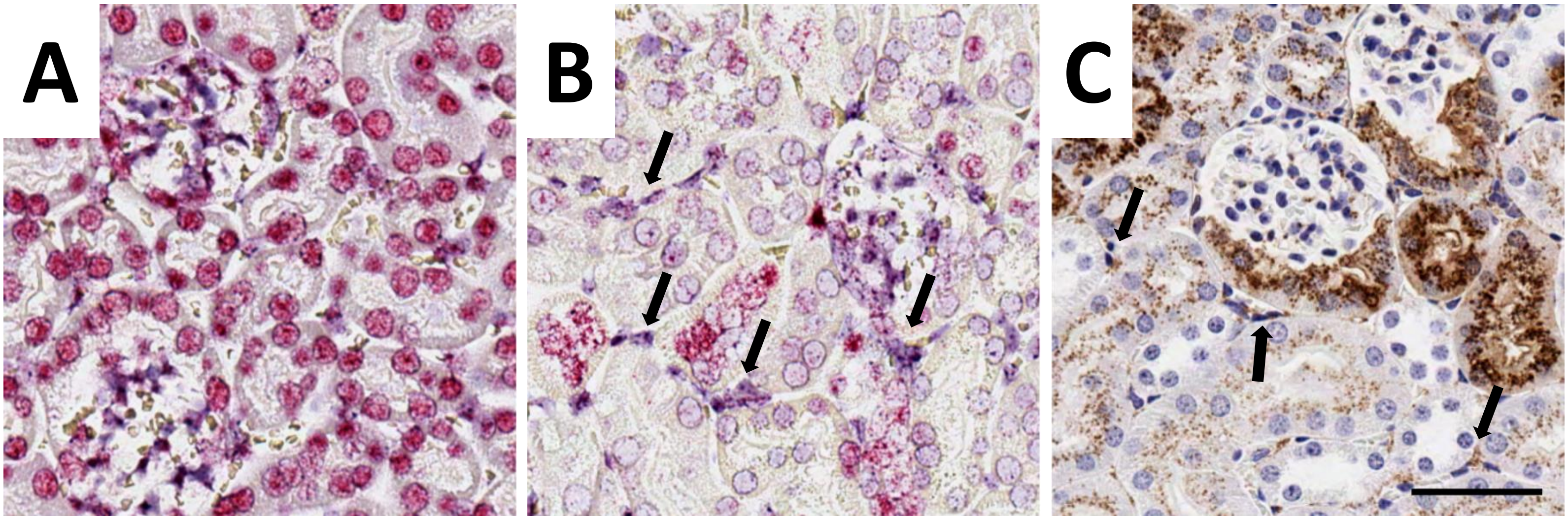

Figure S1

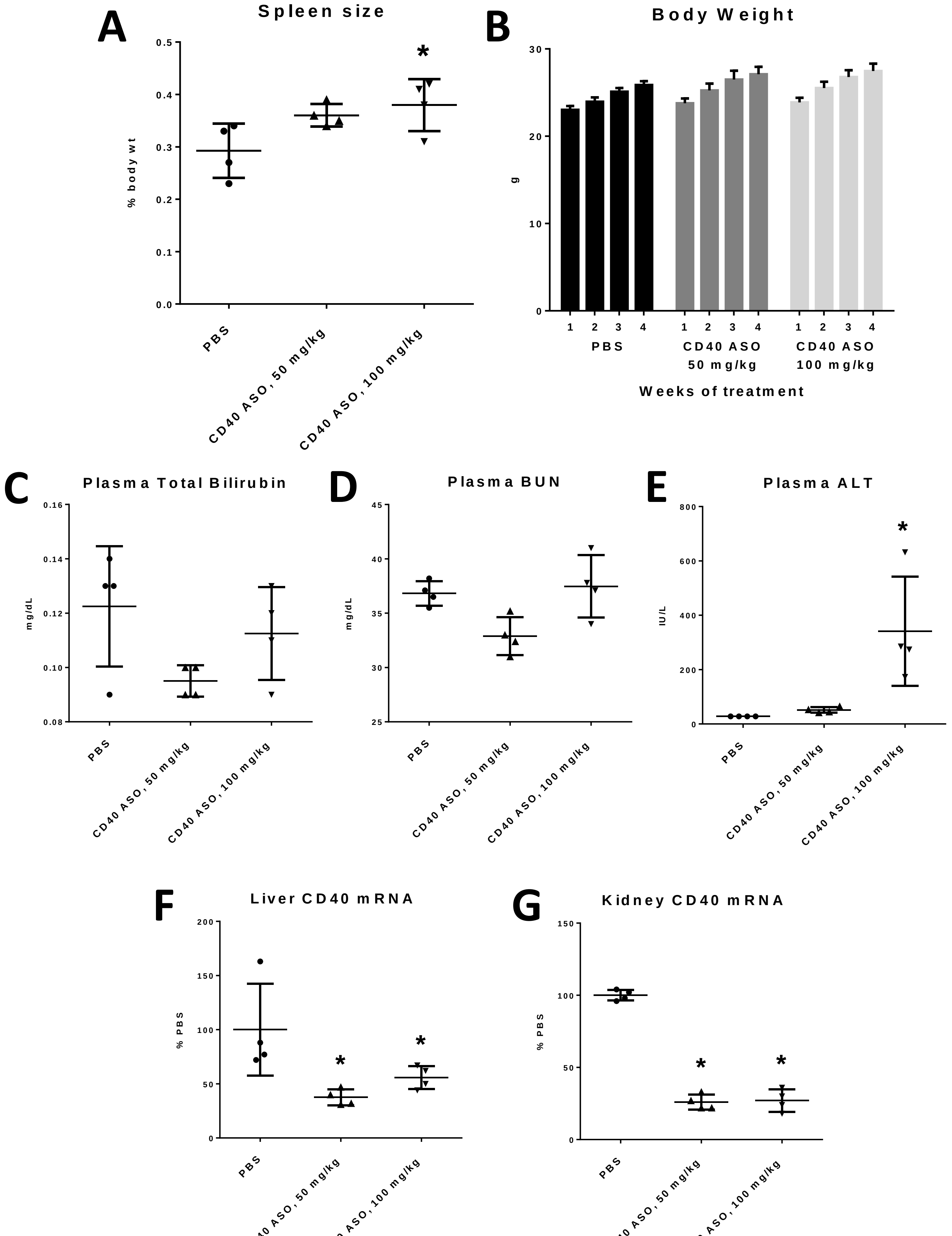
A
Spleen size
% body wt
0.0
0.1
0.2
0.3
0.4
0.5
*
PBS
CD40 ASO, 50 mg/kg
CD40 ASO, 100 mg/kg
B
Body Weight
g
0
10
20
30
1 2 3 4
PBS
CD40 ASO 50 mg/kg
CD40 ASO 100 mg/kg
Weeks of treatment
C
Plasma Total Bilirubin
mg/dL
0.08
0.10
0.12
0.14
0.16
D
Plasma BUN
mg/dL
25
30
35
40
45
E
Plasma ALT
IU/L
0
200
400
600
800
F
Liver CD40 mRNA
% PBS
0
50
100
150
200
G
Kidney CD40 mRNA
% PBS
0
50
100
150

Figure S2

A

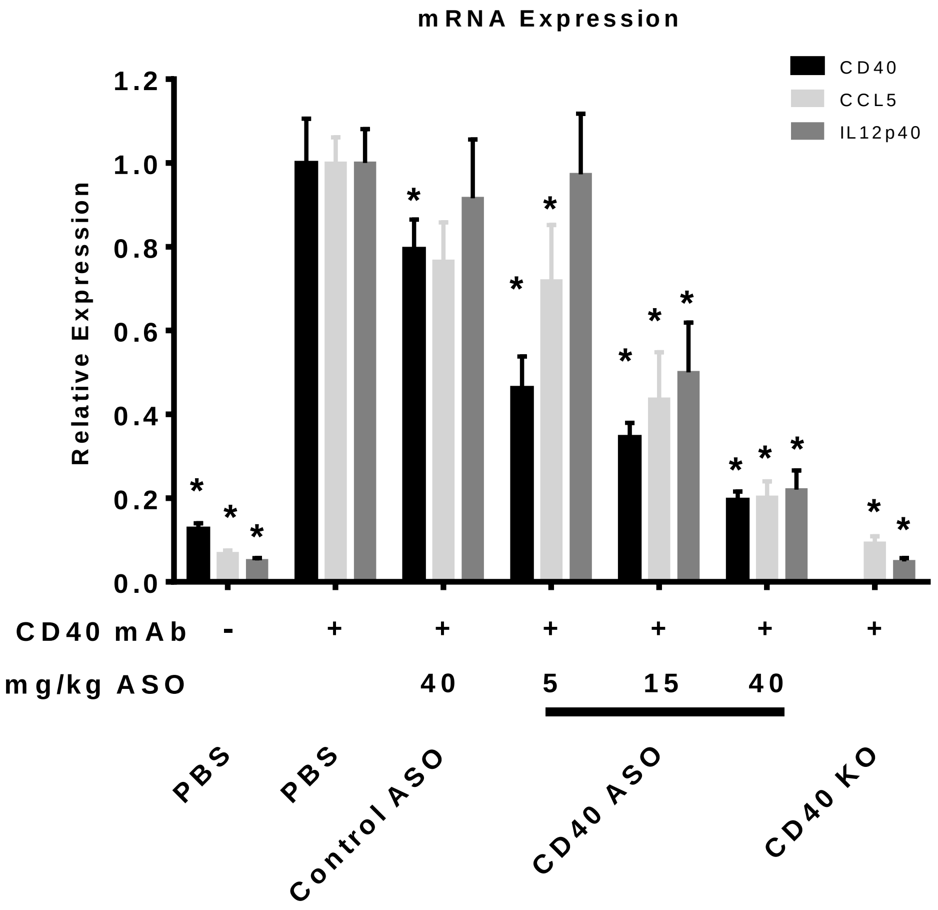
mRNA Expression
CD40
CCL5
IL12p40
Relative Expression
1.2
1.0
0.8
0.6
0.4
0.2
0.0
CD40 mAb - + + + + + +
mg/kg ASO 40 5 15 40
PBS
PBS
Control ASO
CD40 ASO
CD40 KO

B

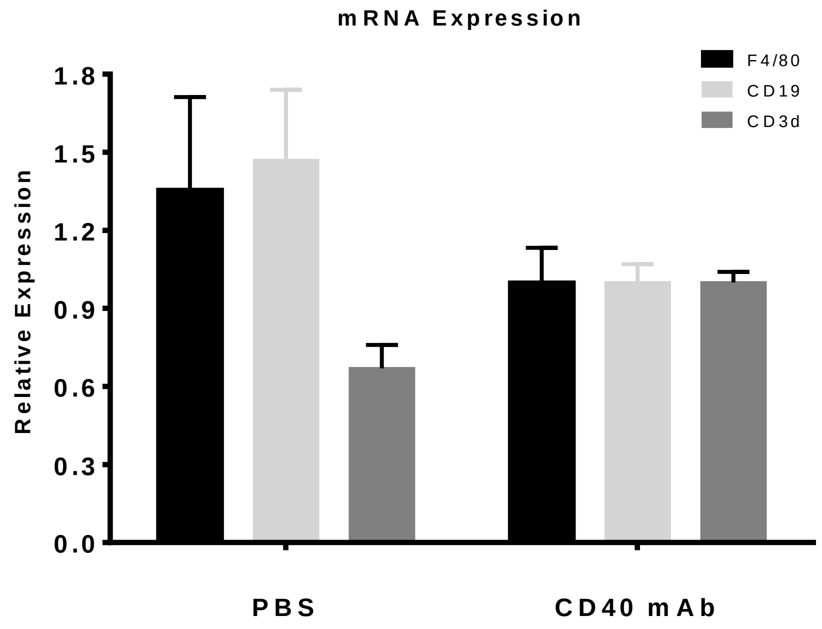
mRNA Expression
F4/80
CD19
CD3d
Relative Expression
1.8
1.5
1.2
0.9
0.6
0.3
0.0
PBS
CD40 mAb

Figure S3

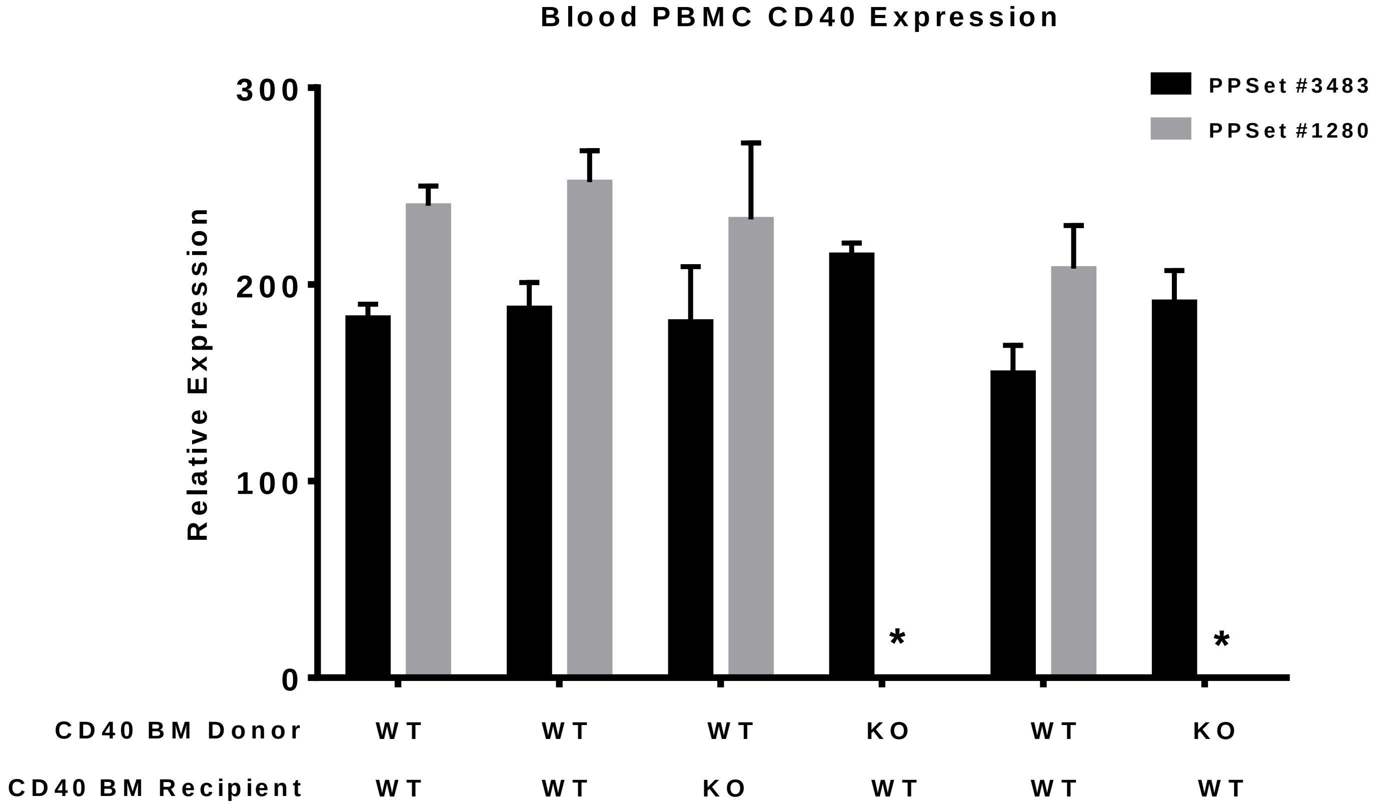

*P < 0.05 vs. WT/WT

Figure S4

Fold-change mRNA levels ± SEM (§ except CD3d IHC), relative to PBS Dox animals

	Dox				No Dox
	PBS	Con ASO-25	CD40 ASO-12.5	CD40 ASO-25	PBS
CD3d	1.00 ± 0.18	0.86 ± 0.11	0.87 ± 0.11	0.61 ± 0.04*	0.56 ± 0.04*
CD4	1.00 ± 0.12	0.86 ± 0.14	0.90 ± 0.14	0.57 ± 0.08*	0.36 ± 0.03*
CD8b	1.00 ± 0.13	0.90 ± 0.15	0.90 ± 0.15	0.56 ± 0.08*	0.25 ± 0.01*
IL-2	1.00 ± 0.12	0.58 ± 0.11*	0.60 ± 0.11*	0.34 ± 0.04*	0.24 ± 0.05*
CD3d IHC§	40 ± 23	37 ± 23	35 ± 24	24 ± 12	24 ± 8
CD68	1.00 ± 0.18	0.99 ± 0.13	1.26 ± 0.17	1.10 ± 0.10	0.34 ± 0.02*
IL-12	1.00 ± 0.12	0.75 ± 0.14	0.91 ± 0.08	0.91 ± 0.11	0.38 ± 0.03*
TNF-α	1.00 ± 0.17	0.69 ± 0.10	0.93 ± 0.08	0.80 ± 0.07	0.24 ± 0.01*
TGF-β	1.00 ± 0.08	0.94 ± 0.08	1.22 ± 0.06*	1.00 ± 0.05	0.67 ± 0.06*
CTGF	1.00 ± 0.06	0.97 ± 0.11	0.69 ± 0.05*	0.73 ± 0.05*	0.46 ± 0.04*
CD206	1.00 ± 0.16	0.95 ± 0.11	0.88 ± 0.08	0.86 ± 0.15	0.45 ± 0.04*

$^*P < 0.05$ vs. PBS (DOX)

Table S1

Sequence information for the probe and primers used to generate the RT-PCR data
*** Sequence information not available from manufacturer (Life Technologies). Unique identifier listed.**

Target mRNA	Forward Primer	Reverse Primer	Probe
**CCL5**	GCAGTCGTGTTTGTCACTCGAA	GATGTATTCTTGAACCCACTTCTTCTC	AACCGCCAAGTGTGTGCCAACCC
**CD206**	GAATACACAGCACTAGCGTCTTAACAC	GGGCTTACGTGGTTGTTTCTAGA	TAGGTCTCATGTCAACCCTGCAGATTTCAAG
**CD3d**	GAGGTTTCTCAAAGTGCCATAGC	CGGAAGGAAACGTCTTTATTGG	ACGGCGCCTTCCCCTGTGATC
**CD4**	Mm00442754*		
**CD40 (#3483)**	CCATCTAGGGCAGTGTGTTACG	CCTGGCTGGCACAAATCAC	ACAAACAGTACCTCCACGATGGCCAGTG
**CD40 (#1280, to genotype the CD40 KO)**	AGCCAGGAAGCCGACTGA	CCCTTGATTGGGTTCACAGTGT	ACTCAGGCGAATTC
**CD40L**	Mm00441911*		
**CD68**	TTGGGAACTACACGTGGGC	CGGATTTGAATTTGGGCTTG	AACGGCTCCCAGCCTTGTGTTCAG
**CD8b**	Mm00438116*		
**COL1A1**	TGGATTCCCGTTCGAGTACG	TCAGCTGGATAGCGACATC	AAGCGAGGGCTCCGACCCGA
**E Selectin**	Mm01310197*		
**F4/80**	GGCCATTGCCCAGATTTTC	CGGTTGAGCAGACAGTGAATGA	CCAGATTGGCCCCTTGGCAAGC
**GAPDH**	GGCAAATTCAACGGCACAGT	GGGTCTCGCTCCTGGAAGAT	AAGGCCGAGAATGGGAAGCTTGTCATC
**ICAM1**	CCGCAGGTCCAATTCACACT	CAGAGCGGCAGAGCAAAAG	CAGCTCGGAGGATCACAAACGAAGCT
**IL-12p40 (IL12/IL23)**	GCCAGTACACCTGCCACAAA	GACCAAATTCCATTTTCCTTCTTG	AGGCGAGACTCTGAGCCACTCACATCTG
**IL-2**	Mm00434256*		
**MCP1**	AGTTGACCCGTAAATCTGAAGCTAA	CACACTGGTCACTCCTACAGAAGTG	CATCCACTACCTTTTCCACAACCACCTCA
**Nephrin**	Mm01176615*		
**NGAL**	GGCCTCAAGGACGACAACA	ACCACCCATTCAGTTGTCAATG	CATCTTCTCTGTCCCCACCGACCAA
**TGF-β**	AAACGGAAGCGCATCGAA	GGGACTGGCGAGCCTTAGTT	CCATCCGTGGCCAGATCCTGTCC
**CTGF**	Mm01192933_g1		
**TNF-α**	CAGGTTCTGTCCCTTTCACTCACT	CTGTGCTCATGGTGTCTTTTCTG	CCCAAGGCGCCACATCTCCCT
**VCAM1**	GGTGCTGTGACAATGACCTGTT	ATTATCTAACTTCCTGCCCCAGAAAAT	CAGCGAGGGTCTACCAGCTCCTGAGA

Table S2
